# Response Adaptation in Barrel Cortical Neurons Facilitates Stimulus Detection during Rhythmic Whisker Stimulation in Anesthetized Mice

**DOI:** 10.1523/ENEURO.0471-18.2019

**Published:** 2019-04-02

**Authors:** Natali Barros-Zulaica, Alessandro E. P. Villa, Angel Nuñez

**Affiliations:** 1 Departamento de Anatomía, Histología y Neurociencia, Facultad de Medicina, Universidad Autónoma de Madrid, Madrid 28029, Spain; 2 Blue Brain Project; École Polytechnique Fédéral de Lausanne, 1015 Lausanne, Switzerland; 3 Laboratoire de Neuroheuristique, Groupe de Recherche en, Science de la Complexité, Université de Lausanne, 1015 Lausanne, Switzerland

**Keywords:** barrel cortex, IGF-I, NMDA, response adaptation, stimulation pattern, whisker movements

## Abstract

Rodents use rhythmic whisker movements at frequencies between 4 and 12 Hz to sense the environment that will be disturbed when the animal touches an object. The aim of this work is to study the response adaptation to rhythmic whisker stimulation trains at 4 Hz in the barrel cortex and the sensitivity of cortical neurons to changes in the timing of the stimulation pattern. Longitudinal arrays of four iridium oxide electrodes were used to obtain single-unit recordings in supragranular, granular, and infragranular neurons in urethane anesthetized mice. The stimulation protocol consisted in a stimulation train of three air puffs (20 ms duration each) in which the time interval between the first and the third stimuli was fixed (500 ms) and the time interval between the first and the second stimuli changed (regular: 250 ms; “accelerando”: 375 ms; or “decelerando” stimulation train: 125 ms interval). Cortical neurons adapted strongly their response to regular stimulation trains. Response adaptation was reduced when accelerando or decelerando stimulation trains were applied. This facilitation of the shifted stimulus was mediated by activation of NMDA receptors because the effect was blocked by AP5. The facilitation was not observed in thalamic nuclei. Facilitation increased during periods of EEG activation induced by systemic application of IGF-I, probably by activation of NMDA receptors, as well. We suggest that response adaptation is the outcome of an intrinsic cortical information processing aimed at contributing to improve the detection of “unexpected” stimuli that disturbed the rhythmic behavior of exploration.

## Significance Statement

To detect objects, rodents scan the environment by rhythmic movements of whiskers at 4-12 Hz, suggesting that this rhythmic behavior may facilitate stimulus detection. This rhythmic input in the somatosensory cortex will be disturbed when the animal touches an object. We study whether spike responses in the somatosensory cortex are sensitive to small changes in the timing of the rhythmic sensory input. We find that responses were facilitated when a modification of the stimulation interval was introduced in the stimulation sequence. This facilitation was mediated by activation of NMDA receptors and increased during periods of EEG activation induced by IGF-I application. We suggest that the detection mechanism of shifted stimulus may play a role in sensory processing.

## Introduction

Animals actively gather sensory information through self-generated movements. For example, eye movements are used to foveate interesting regions of visual space. Eye movements can determine the visual sensory input that falls on the retina. Active touch is also a common behavior to discern the shape, size, and texture of objects (Szwed et al., 2003; Kleinfeld et al., 2006; Diamond and Arabzadeh, 2013; Bale and Maravall, 2018).

In rodents, tactile discrimination during exploratory behaviors is based on repetitively and rapidly swept back and forth of whiskers (whisking) across objects or surfaces in repeated rhythmic movements at frequencies between 4 and 12 Hz, scanning their surroundings to obtain tactile information about nearby objects (Carvell and Simons, 1990; Berg and Kleinfeld, 2003; Von Heimendahl et al., 2007; Jadhav et al., 2009). Whisking in free air induces spike trains in the somatosensory barrel cortex (BC; Curtis and Kleinfeld, 2009), which will be disturbed when the animal touches an object (for review, see Kleinfeld and Deschênes, 2011). In addition, spike trains suffer a reduction of the response with time in a process termed as response adaptation. The functional role of the response adaptation process may be to alter the sensitivity of neurons to encode more efficiently sensory stimuli (Sharpee et al., 2006; Maravall et al., 2007, 2013; Ganmor et al., 2010) or to improve the detectability of rare stimuli by decreasing responses to frequent stimuli (Dragoi et al., 2002; Ulanovsky et al., 2003). It is established that neuronal responses in the BC adapt robustly to repetitive whisker stimulation (Ahissar et al., 2000, 2001; Chung et al., 2002; Khatri et al., 2009; Martin-Cortecero and Nuñez, 2014). However, response adaptation during whisking may be altered when a change in the interval between stimuli occurs, for example when whiskers touch an object.

The effect of the temporal pattern in a spike train has been studied by Segundo and colleagues (Perkel et al., 1963; Segundo et al., 1963; Villa et al., 2007). Stimulation with triplets in *Aplysia* ganglia showed that neuronal responses were sensitive to the timing of the stimulation pattern (Segundo et al., 1963). These findings suggest that a train of spikes induced, for example, by whisking may contain information according to the temporal pattern of the sensory input. Our intention in this work is to determine whether spike responses in the BC are sensitive to small changes in the timing of the stimulation pattern (temporal jitter). We applied rhythmic whisker stimulation trains of three stimuli at a mean frequency of 4 Hz in which all intervals were equal (“regular” stimulation pattern, 250 ms between stimuli) or varied in a ramp: “accelerando” (intervals decreased, 375 + 125 ms) or “decelerando” (intervals increased, 125 + 375 ms). These stimulation patters try to mimic the sensory input when a whisker touches an object during whisking. Sensitivity to stimulation patterns has a general interest because it contributes to understand how the somatosensory system handles complex temporal information.

## Materials and Methods

## Animals

The experiments were performed on 24 adult C57Bl/6J mice (25–30 g weight) of either sex. Mice were group housed with a 12 h light/dark cycle and had *ad libitum* to food and water. In accordance with European Community Council Directive 2010/63/UE all animal procedures were approved by the Ethical Committee of the Universidad Autónoma de Madrid (CEI72-1286-A156). Efforts were made to minimize animal suffering as well as to reduce the number of animals used.

### Electrophysiological recordings

**Figure 1.**
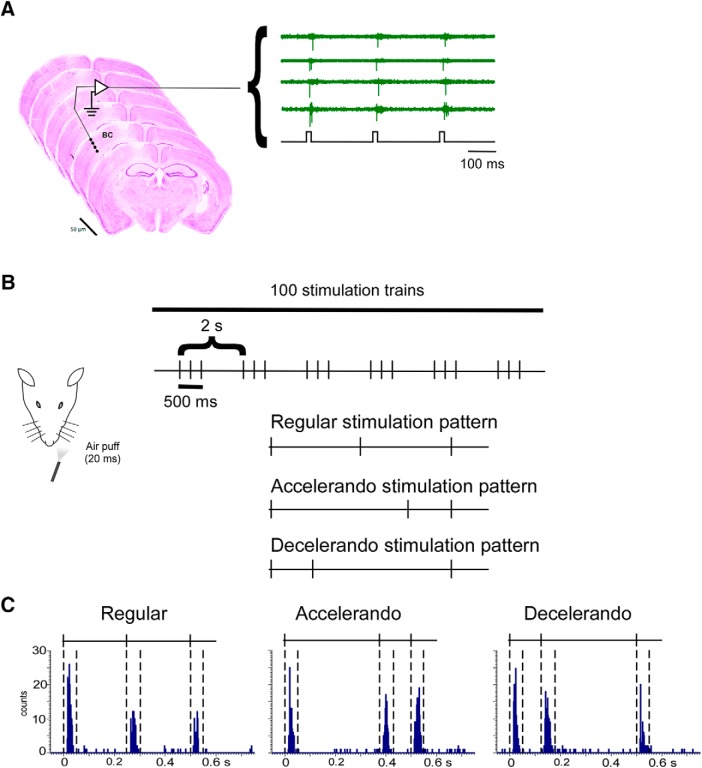
Response adaptation is altered by a change in the stimulation pattern. ***A***, Serial brain slices of a representative case in which the electrode track is observed. An example of spike responses is shown on the right (bottom recording represent stimuli). ***B***, A schematic diagram of the experimental protocol and stimulation patterns. ***C***, Representative PSTHs of a single unit located in layer 5 show response adaptation when a regular stimulation pattern is applied (left PSTH). Response adaptation is reduced if the interval between the first and the second stimuli increases from 250 to 375 ms (accelerando stimulation pattern; middle PSTH) or decreases to 125 ms (decelerando stimulation pattern; right PSTH). Lines on the PSTHs show the stimulation pattern. Vertical dashes lines indicate 50 ms poststimulus time window in which spike responses are calculated. *Figure Contributions:* Natali Barros-Zulaica performed the experiments. All authors analyzed the data.

General anesthesia was induced by peritoneal injection of urethane (1.2 g/kg, i.p.). Depth of anesthesia was sufficient to eliminate pinch withdrawal, palpebral reflex and whisker movement and was assessed periodically throughout the surgical procedure and during electrophysiological recordings. Animals were placed in a Kopf small-animal stereotaxic device (David Kopf Instruments) in which surgical procedures and recordings were performed. The body temperature was maintained at 37°C. An incision was made exposing the skull and a small hole was drilled in the bone in the skull overlying the whisker barrel field of the somatosensory BC (A: 0–2 mm, L: 3–4 mm from bregma and V: 0.3–1.1 μm from the dura mater; Paxinos and Franklin, 2003). Longitudinal arrays of four Iridium oxide electrodes (15 μm electrode diameter; 200 μm separation between electrodes; 1–2 MΩ impedance; Q1x4-5mm-200-177-Q4) manufactured by NeuroNexus Technologies were used to obtain single-unit recordings in the BC. The array was carefully advanced perpendicular to the cortical surface, using a remote-controlled micromanipulator (David Kopf Instruments). Thus, the most superficial recording site was located at layer 2/3 (300 μm from the surface) and the deepest one was located at layer 6 (1100 μm from the surface; Fig. 1*A*).

Extracellular signals were preamplified through a home-made preamplifier. The signals were further amplified and bandpass filtered between 300 Hz and 3 kHz using an AC preamplifier (1902 isolated preamplifier; Cambridge Electronic Design), digitized at 10 kHz (Power 1401, CED), and stored for off-line analysis. The recording session lasted for 4–5 h after the beginning of the recording procedure.

### Whisker stimulation

For each recording, the principal whisker was determined as the whisker that caused the strongest response to manual mechanical deflection. All selected neurons responded to passive whisker deflections using a hand-held probe or air-puff stimuli generated by a pneumatic pressure pump (Picospritzer). After identification of the receptive field with the handheld probe, air-puff stimulation was delivered by aiming a 1 mm inner diameter polyethylene tube 10–20 mm away from the receptive field so that whiskers were pushed backward by a 20 ms duration puff (20–30 psi).

The stimulation protocol consisted in a stimulation train of three air puffs in which the time interval between the first and the third air puff was fixed (500 ms) and the time interval between the first and the second air puff changed (Fig. 1*B*):

- *Regular stimulation pattern*: the time interval between stimuli was 250 ms.
- *Accelerando stimulation pattern*: the interval between the first and the second stimuli was 375 ms and thus, the second and the third stimuli were closer (125 ms).
- *Decelerando stimulation pattern*: the interval between the first and the second stimuli was 125 ms and thus, the second and the third stimuli were 375 ms faraway.

In all cases the mean frequency of the stimulation train was 4 Hz and was repeated 100 times at 0.5 Hz (stimulation block). These stimulation protocols tried to compare a regular stimulation pattern at 4 Hz with a stimulation pattern containing temporal jitter in the second stimuli. This pattern of stimulation is reminiscent of that produced by an object that interferes with the rhythmic movement of whiskers during the whisking behavior.

## Drugs

The following drugs were used: 2-amino-5-phosphonopentanoic acid (AP5; 50 μm), which is a selective NMDA (Sigma-Aldrich Quimica) receptor antagonist. AP5 was injected (0.1 μl) through a cannula connected to a Hamilton syringe located 300 μm deep in the BC to affect the entire cortical column. IGF-I (1 μg/g body weight; Peprotech) was injected intraperitoneally to increase cortical excitability.

## Histology

Following the recording session, electrolytic lesions were performed using five pulses of 5 μA for 10 s at intervals of 10 s at the top and at the bottom of the electrode array for subsequent histologic reconstruction of the electrode tracks in coronal sections through the barrel cortex. At the end of the experiment the brain was processed for histology to corroborate the electrode track and electrode location. Animals were euthanized with an overdose of pentobarbital and perfused through the heart with 100 ml of 0.9% NaCl, followed by 100 ml 4% paraformaldehyde in 0.1 m phosphate buffer. Coronal sections (50 μm) were obtained using a Leica freezing microtome and stained with cresyl violet (Fig. 1*A*).

### Statistical analysis

One to two cells were detected from each single electrode based on the spike amplitude and wave form. Selected neurons showed a response to the stimulation of a whisker located in the contralateral pad. Only neurons that met this criterion were used for further analysis. According to their firing rate of the recorded neurons we assumed that all recorded neurons were pyramidal (see Results section). Spikes were digitally stored for time series analysis. All subsequent analysis was performed with Spike 2 software (Cambridge Electronic Design).

Spontaneous spike firing was calculated from periods of 120 s without whisker stimulation. Peristimulus time histograms (PSTHs) were calculated for cortical neurons, where time 0 ms of the histogram corresponded to the onset for the first stimulus in the stimulation train. Spike responses were calculated in a 50 ms poststimulus time window following each stimulus in the stimulation train (PSTH; 1 ms bin-width; 100 stimulation trains; Fig. 1*C*). The response for the first stimulus of the stimulation train was considered 100%; the response for the second and the third stimuli was calculated with respect to this one. These response percentages were calculated in each neuron and the average and the SEM are indicated in the text and figures. Also, PSTHs were calculated for the first or for the last 25 stimulation trains of the 100 stimulation train block. Response duration was defined as the time elapsed from the onset to offset responses; response onset was defined as the first of three consecutive bins displaying significant activity (2× higher than the mean firing rate) after stimulus and response offset as the last bin of the last three consecutive bins displaying significant activity. Response duration was measured for each stimulus into the stimulation train for each of the different stimulation patterns.

Statistical analysis was performed using GraphPad Prism 7 software. Statistical analyses consisted, for the most part, of paired comparisons of responses evoked in the same neuron by different stimulation trains or drugs. Data were considered normally distributed, according to the Shapiro–Wilk normality test, and thus, we used parametric statistics. We used the *t* test (paired or independent) to compare data from two groups. The statistic test was applied on the values of response (spikes/stimulus; Table 1) in different conditions. Differences were considered statistically significant at the 95% level (*p* < 0.05). However, the normalized percentage respect to the first response in the stimulation train is shown in Figures and the statistical significant is indicated with asterisks. Data are presented as mean ± SEM.

**Table 1.** Cortical responses to the whisker stimulation train (St. 1–3) according to the stimulation pattern (spikes/stimulus)

**Layer**	**Regular**			**Accelerando**			**Decelerando**		
	**St. 1**	**St. 2**	**St. 3**	**St. 1**	**St. 2**	**St. 3**	**St. 1**	**St. 2**	**St. 3**
Supragranular	1.37 ± 0.26 *n* = 14	0.89 ± 0.18 *n* = 14	0.69 ± 0.1 *n* = 14	1.26 ± 0.20 *n* = 14	1.17 ± 0.23 *n* = 14	1.07 ± 0.29 *n* = 14	1.56 ± 0.20 *n* = 14	1.55 ± 0.26 *n* = 14	1.12 ± 0.15 *n* = 14
Granular	1.26 ± 0.16 *n* = 12	1.17 ± 0.16 *n* = 12	0.99 ± 0.1 *n* = 12	1.56 ± 0.25 *n* = 12	1.84 ± 0.27 *n* = 12	1.87 ± 0.37 *n* = 12	1.49 ± 0.25 *n* = 12	1.83 ± 0.3 *n* = 12	1.44 ± 0.25 *n* = 12
Infragranular	1.88 ± 0.29 *n* = 23	1.33 ± 0.13 *n* = 23	1.18 ± 0.1 *n* = 23	1.77 ± 0.19 *n* = 23	1.66 ± 0.19 *n* = 23	1.73 ± 0.25 *n* = 23	1.94 ± 0.18 *n* = 23	1.81 ± 0.2 *n* = 23	1.42 ± 0.17 *n* = 23

## Results

To study the effect of different stimulation patterns in BC responses we separated recorded cortical cells into three groups according to the cortical depth and observation of the histologic preparations (Fig. 1*A*): supragranular cells (up to 500 μm below the pia; layers 2–3); granular cells between 500 and 800 μm approximately representing layer 4 cells and infragranular cells below 800 μm (layers 5–6). Neurons recorded in the BC (*n* = 66) showed a low spontaneous firing rate as is characteristic of the urethane anesthetized mice (de Kock and Sakmann, 2009). Granular and supragranular neurons showed the lowest spontaneous firing rate 0.9 ± 0.22 spikes/s (*n* = 16) and 1.0 ± 0.22 spikes/s (*n* = 16), respectively, whereas infragranular neurons showed higher firing rate, 1.7 ± 0.18 spikes/s (*n* = 34). The low spontaneous firing rate strongly suggests that these recordings could be performed in pyramidal dendrites because they have firing rates larger than somatic spike rates (Moore et al., 2017). Results are based on the analysis of BC neurons which responded to whisker stimulation of the contralateral pad. There were no gender-related differences in the results; for this reason, they were pooled together. Responses consisted in a short-latency spike-burst of 1–3 action potentials. Supragranular neurons showed 1.4 ± 0.26 spikes/stimulus (*n* = 16), granular neurons 1.3 ± 0.16 spikes/stimulus (*n* = 16), and infragranular neurons 1.9 ± 0.29 spikes/stimulus (*n* = 34), measured in a 50 ms poststimulus time window following the stimulus onset.

### The stimulation pattern altered response adaptation

**Figure 2.**
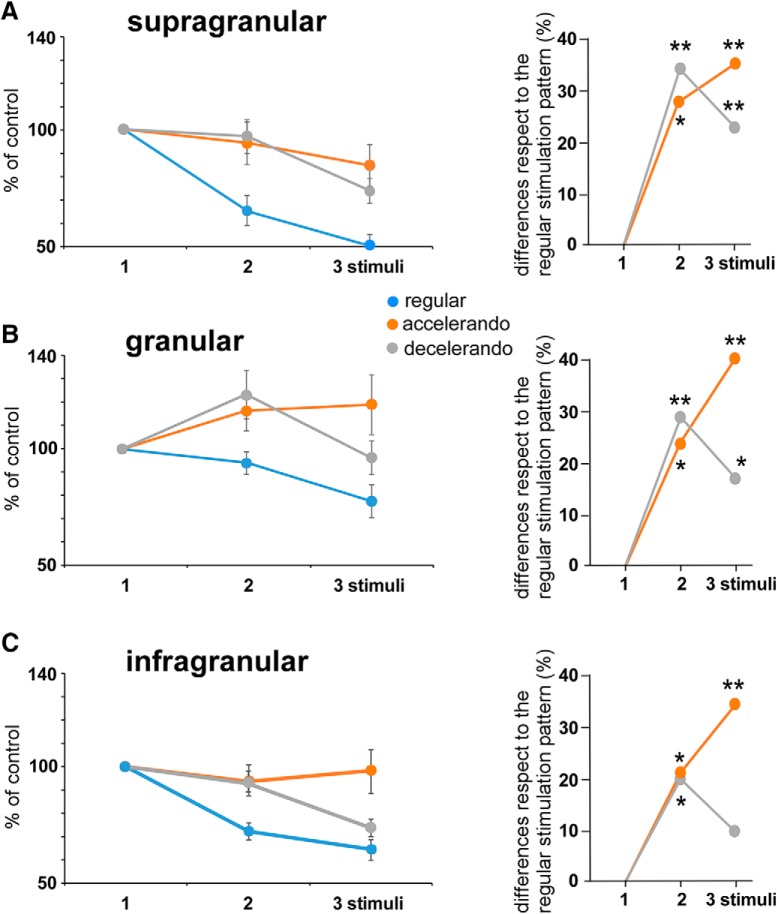
Response adaptation is altered by a change in the stimulation pattern. ***A***, Supragranular neurons (*n* = 14) show a reduction in spike response during the application of the regular stimulation pattern (three stimuli at 4 Hz; blue circles). The mean percentage of the response respect to the control stimulus (first stimulus of the stimulation train) is shown on the left. The response adaptation is reduced if the interval between the first and the second stimuli increases from 250 to 375 ms (accelerando stimulation pattern; orange circle) or decreases to 125 ms (decelerando stimulation pattern; gray circle). The plots shown on the right display differences in the percentage of responses to the 1–3 stimuli during the application of the accelerando or decelerando stimulation patterns respect to the corresponding percentage to the 1–3 stimuli during the application of a regular stimulation pattern. In all cases the response to the second and third stimuli is higher than is expected during the regular stimulation pattern. ***B***, ***C***, Same plots as in ***A*** from granular (*n* = 12) and infragranular (*n* = 23) neurons, respectively. Equally, responses during the accelerando or decelerando stimulation pattern are greater respect to the corresponding response during the application of a regular stimulation pattern. *p < 0.05; **p < 0.01. *Figure Contributions:* Natali Barros-Zulaica performed the experiments. All authors analyzed the data.

Cortical neurons adapted strongly their response to regular stimulation trains at 4 Hz, as shown by the PSTHs. A representative example of a neuron located in layer 5 is shown in Figure 1*C* (left histogram) in which the spike response decreased during the stimulus train. The population analysis indicated that adaptation was lower in the granular layer than in supragranular or infragranular layers (Table 1, regular stimulation pattern; Fig. 2, blue circles in all figures), consistent with other previous studies (Higley and Contreras, 2003; Khatri et al., 2004; Martin-Cortecero and Nuñez, 2014). The response adaptation was reduced if the interval between the first and the second stimulus increased from 250 to 375 ms (accelerando stimulation pattern; orange circles in all figures) or decreased to 125 ms (decelerando stimulation pattern; gray circles in all figures). Examples of these differences are depicted in Figure 1*C*, where PSTHs were calculated from a unit located in layer 5 when different stimulation patterns were applied. The response to the second and third stimuli of the stimulation train varied according to the interval between stimuli. Figure 2 shows population data measuring the mean percentage of the response respect to the control (first stimulus of the stimulation train; 100%) for each cortical layer (*A*–*C*, supragranular, granular or infragranular layers, respectively); plots of responses to the 1–3 whisker stimuli are shown on the left. Differences between one and three stimulus responses during the application of the accelerando or decelerando stimulation pattern relative to the corresponding response during the application of a regular stimulation pattern are shown on the right. In all cases the response to the second and third stimuli was higher than during the application of the regular stimulation pattern, indicating that the adaptation observed in all layers during the application of the regular stimulation pattern was diminished when the interval between stimuli changed.

The response to the second and third stimuli in supragranular neurons (*n* = 14) was higher during the application of the accelerando stimulation pattern comparing to the regular stimulation pattern (28 and 35%, respectively; *p* = 0.045 and *p* = 0.0022, respectively; paired *t* test; Fig. 2*A*; Table 1) as well as during the application of the decelerando stimulation pattern (34 and 22%, respectively; *p* = 0.0022 and *p* = 0.0014, respectively; paired *t* test). Equally, this effect was observed in granular neurons (*n* = 12). Responses were higher during the application of the accelerando stimulation pattern (24 and 41%, respectively; *p* = 0.0192 and *p* = 0.0019; paired *t* test) or the decelerando stimulation pattern than it was expected during the application of the regular stimulation pattern (29 and 18%, respectively; *p* = 0.0029 and *p* = 0.019; paired *t* test; Fig. 2*B*; Table 1).

Neurons in infragranular layers also altered their response when a change of the interval between the first and the second stimuli occurred, especially during the application of the accelerando stimulation pattern. Layer 5 neurons (*n* = 23) showed a higher response during the application of the accelerando stimulation patter than it was expected during the application of the regular stimulation pattern (21 and 35%, for the second and third stimuli of the train, respectively; *p* = 0.0103 and *p* = 0.0005, respectively; paired *t* test; Fig. 2*C*; Table 1). During the application of the decelerando stimulation pattern only the response to the second stimulus was higher than the response during the regular stimulation pattern (20%; *p* = 0.0181; paired *t* test; Fig. 2*C*; Table 1); the response to the third stimulus was not statistically different (10%; *p* > 0.05). In conclusion, the above results indicated that cortical cells are able to detect a change in the stimulation pattern, facilitating the detection of no-regular timing stimuli that appear into a rhythmic sequence of stimulation.

**Figure 3.**
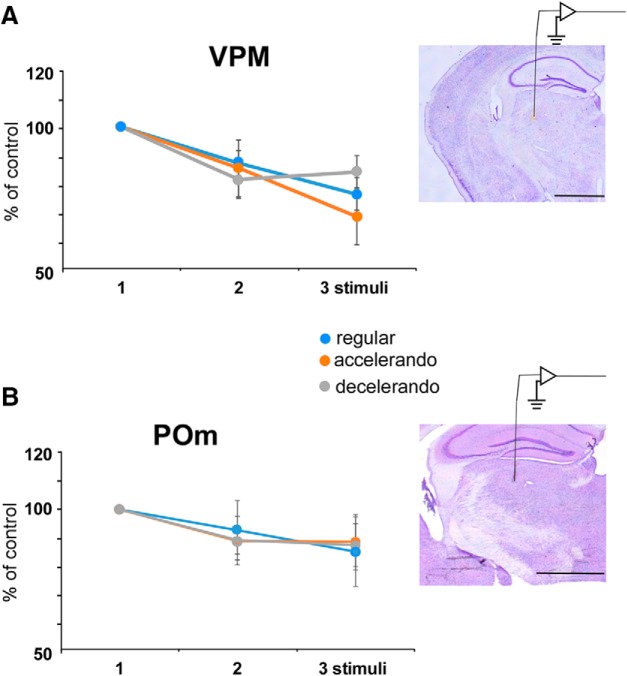
Response adaptation in thalamic neurons is not altered by the stimulation pattern. ***A***, The mean percentage of the response respect to the control stimulus (first stimulus of the stimulation train) is shown for VPM (*n* = 12). ***B***, Same plot that in A for POm (*n* = 8) neurons. Response adaptation is not altered by the stimulation pattern (regular, accelerando or decelerando stimulation patterns. Right, Brain slices of representative cases in which the electrode track is observed. Scale bars, 1 mm. *Figure Contributions:* Natali Barros-Zulaica performed the experiments. All authors analyzed the data.

To establish whether this mechanism is proper of the cortex or appears in other subcortical relay stations of the somatosensory pathway we recorded multiunit activity in the ventro-posterior (VPM) or posterior-medial (POm) thalamic nuclei. VPM recordings showed that response adaptation was the same when VPM neurons were stimulated with the regular, accelerando or decelerando stimulation patterns (*n* = 12; Fig. 3*A*). The same occurred when POm thalamic neurons were recorded (*n* = 8; Fig. 3*B*).

**Figure 4.**
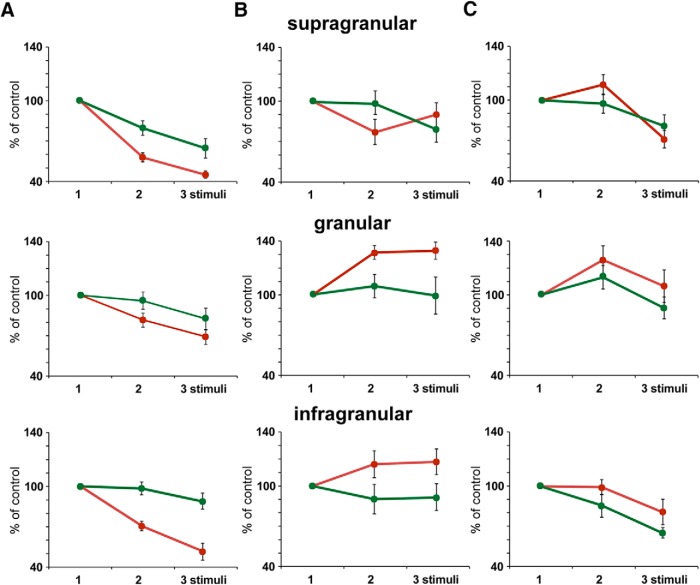
Response adaptation is altered during the stimulation period. Plots of the mean percentage of the response respect to the control stimulus (first stimulus of the stimulation train; 100%) are shown. Data are calculated from PSTHs calculated with the first 25 stimulation trains (red) or with the last 25 stimulation trains (green) of the 100 stimulation train block applied to BC neurons. ***A***, Response adaptation during the application of the regular stimulation pattern is larger in the first 25 stimulation trains for supragranular (*n* = 14; top plots), granular (*n* = 12; middle plots), and infragranular (*n* = 23; bottom plots) neurons. ***B***, ***C***, Same plots that in ***A*** for neurons recorded during the application of accelerando or decelerando stimulation patterns, respectively. In all cases the response to the second and third stimuli is higher in the first stimulation trains than in the last stimulation train of the stimulation block. *Figure Contributions:* Natali Barros-Zulaica performed the experiments. All authors analyzed the data.

It is expected that the detection of the stimulus may change over time during the application of successive trains of stimuli. Above findings in the BC show the mean response after the application of a block of 100 stimulation trains. It is logical to believe that these findings are not the same during the long-lasting stimulation train. Figure 4 compares the response of the first with the last 25 stimulation trains during the application of the 100 stimulation trains with regular, accelerando or decelerando stimulation patterns. In all layers, response adaptation during the application of the regular stimulation pattern was larger during the first stimulation trains (red points) in comparison with the last stimulation trains (Fig. 4*A*, green points). Equally, the facilitation observed when the interval between the first and the second stimuli was longer, during the application of the accelerando stimulation pattern respect to the responses during the regular stimulation pattern, was also larger in granular and infragranular neurons (Fig. 4*B*). However, supragranular neurons showed facilitation respect to the responses observed during the application of the regular stimulation pattern between the second and the third stimuli for the first 25 stimulation trains which disappeared for the last stimulation trains. During the application of the decelerando stimulation pattern the response facilitation observed between the first and the second stimuli was larger in all layers in the first stimulation trains than in the last stimulation trains (Fig. 4*C*). Thus, facilitation processes evoked by a change in the interval between stimuli decayed during the stimulation period. The response adaptation was similar in the last stimulation trains during the application of the regular, accelerando or decelerando stimulation pattern for neurons of all layers.

### NMDA receptors are involved in the detection of a change in the stimulation pattern

**Figure 5.**
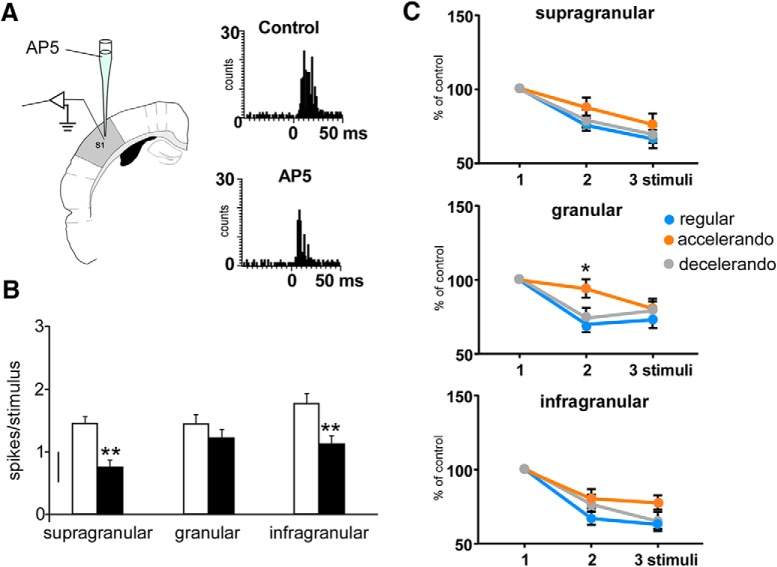
Changes in the response adaptation induced by the stimulation pattern are because of activation of NMDA receptors. ***A***, Schematic diagram of the experimental protocol. Insets, PSTHs of the response of layer 5 neurons in the control condition and 10 min after AP5 application (50 μm, 0.1 μl; black bars); the response is clearly reduced. ***B***, Responses to whisker stimuli are reduced after cortical injection of AP5 (50 μm, 0.1 μl; black bars) respect to control values (white bars) in supragranular (*n* = 8), granular (*n* = 7), and infragranular (*n* = 8) neurons. ***C***, Mean percentage of the response respect to the control stimulus (first stimulus of the stimulation train) is shown. Supragranular and infragranular neurons adapt equally in presence of AP5 when the regular, accelerando, or decelerando stimulation patterns are applied. Granular neurons also show response adaptation during the application of the regular and decelerando stimulation patterns after AP5 application. However, a significant reduction of adaptation is observed during the application of the accelerando stimulation pattern. *p < 0.05; **p < 0.01. *Figure Contributions:* Angel Nuñez performed the experiments. All authors analyzed the data.

Above results indicated that response facilitation occurred when the interval between stimuli was reduced from 250 to 125 ms. Paired-pulse facilitation is because of the activation of NMDA-mediated responses in many cortical regions (Thomson, 2000; Dan and Poo, 2004; Citri and Malenka, 2008). We have investigated the role of this glutamatergic receptor in the detection of the stimulation pattern by the application of the specific NMDA receptor blocker AP5 in the BC (Fig. 5*A*). Application of AP5 through a cannula located next to the recording microelectrode in the BC (50 μm; 0.1 μl; recordings performed 10 min later) decreased tactile responses of supragranular neurons from 1.5 ± 0.19 to 0.7 ± 0.09 spikes/stimulus (*p* < 0.001; *n* = 8; paired *t* test) and from 1.8 ± 0.13 to 1.1 ± 0.09 spikes/stimulus (*p* = 0.005; *n* = 8; paired *t* test) in infragranular neurons. However, granular neurons were not significantly affected (from 1.5 ± 0.21 to 1.2 ± 0.10 spikes/stimulus; *p* > 0.05; *n* = 7; paired *t* test; Fig. 5*B*). A representative example is shown in the PSTHs of Figure 5*A*. AP5 reduced whisker responses, decreasing response duration (see Fig. 7*C*).

Supragranular (*n* = 8) and infragranular (*n* = 8) neurons showed response adaptation during the application of the regular stimulation pattern 10 min after AP5 application (Fig. 5*C*, blue circles). They also adapted in presence of AP5 when the accelerando or decelerando stimulation pattern was applied (Fig. 5*C*, orange or gray circles, respectively); facilitation of the second and the third response was not observed, indicating that response facilitation described above elicited by a change in the interval between stimuli was because of the activation of NMDA receptors. Granular neurons also showed response adaptation during the application of the regular and decelerando stimulation patterns after AP5 application. However, a significant reduction of adaptation was observed during the application of the accelerando stimulation pattern in presence of AP5 (*p* = 0.0325; *n* = 7; paired *t* test; Fig. 5*C*, orange circles), suggesting that other mechanisms may be involved.

**Figure 6.**
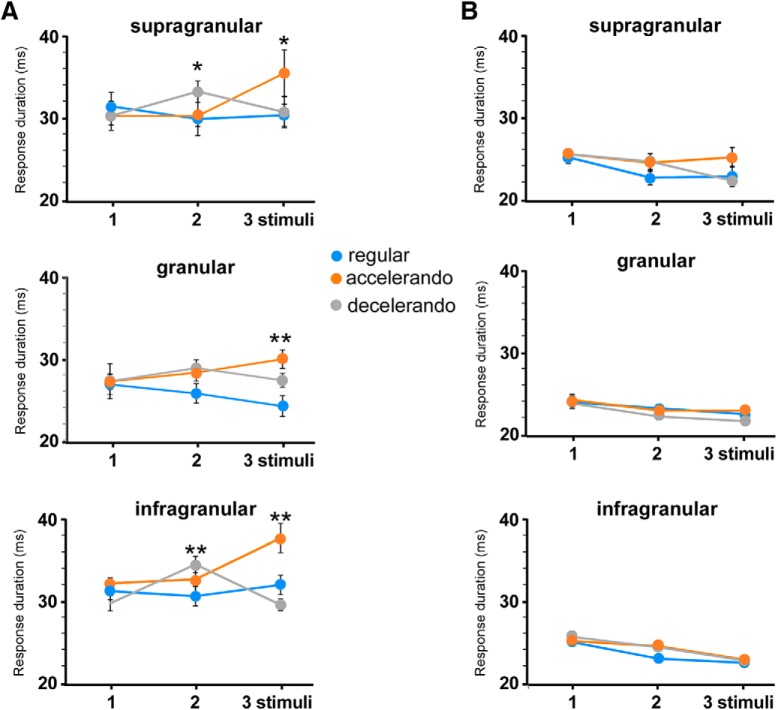
The stimulation pattern also induces changes in the whisker response duration. ***A***, Response duration according to the stimulation pattern in each cortical layer. ***B***, Application of AP5 into the BC (50 μm; 0.1 μl) reduces the duration of whisker responses and blocks the effect of the stimulation pattern. *p < 0.05; **p < 0.01. *Figure Contributions:* Natali Barros-Zulaica and Angel Nuñez performed the experiments. All authors analyzed the data.

In a previous work we have demonstrated that an increase of NMDA-mediated response induces an increase in response duration of the BC neurons (Barros-Zulaica et al., 2014). Thus, we measured the response duration for each stimulus into the stimulation train during the application of different stimulation patterns (see Materials and Methods) to further study the participation of NMDA receptors in the detection of the stimulation pattern. The response duration of granular cells (27.0 ± 1.2 ms) was significantly shorter than responses in supragranular (31.8 ± 1.5 ms; *p* = 0.0211; *t* test) and infragranular neurons (31.1 ± 1.0 ms; *p* = 0.0151; *t* test) when the response duration was measured in the first stimulus of the stimulation train. Figure 6*A* shows the mean response duration of neurons located in supragranular (*n* = 11), granular (*n* = 11), and infragranular layers (*n* = 16) during the application of the regular, accelerando or decelerando stimulation patterns (Table 2). When the interval between stimuli was reduced to 125 ms (between the second and the third stimuli in the accelerando stimulation pattern or between the first and the second stimuli in the decelerando stimulation pattern) the duration increased in the second one, reaching statistical significance in supragranular and infragranular neurons. Granular neurons only showed significant differences during the application of the accelerando stimulation pattern. These findings support that the response facilitation observed during the application of the accelerando or decelerando stimulation pattern was mainly because of activation of NMDA receptors. Accordingly, the application of AP5 into the BC (50 μm; 0.1 μl) reduced the duration of whisker responses in supragranular (from 30.5 ± 1.3 to 25.6 ± 0.7 ms; *n* = 8; *p* = 0.0012; paired *t* test) and in infragranular neurons (from 32.3 ± 1.3 to 25.3 ± 0.5 ms; *n* = 8; *p* < 0.001; paired *t* test). Granular neurons slightly decreased their response duration but did not reach statistical significance (from 26.5 ± 1.5 to 23.9 ± 0.7 ms; *n* = 7; *p* > 0.05; paired *t* test). Furthermore, response duration was not altered by the stimulation pattern in presence of AP5 (Fig. 6*B*). These findings matched above results obtained when spike responses were measured (Fig. 5), indicating that shortening the interval between stimuli in a stimulation train generated a response facilitation that was mediated by the activation of NMDA receptors.

**Table 2.** Duration of cortical responses to whisker stimulation trains of three stimuli (St. 1–3), according to the stimulation pattern (in ms)

**Layer**	**Regular**			**Accelerando**			**Decelerando**		
	**St. 1**	**St. 2**	**St.3**	**St. 1**	**St. 2**	**St. 3**	**St. 1**	**St. 2**	**St. 3**
Supragranular	31.8 ± 1.5 *n* = 11	29.8 ± 0.9 *n* = 11	30.3 ± 1.3 *n* = 11	30.2 ± 1.8 *n* = 11	30.2 ± 2.4 *n* = 11	35.5 ± 2.9 *n* = 11	30.2 ± 1.1 *n* = 11	33.2 ± 1.3 *n* = 11	30.6 ± 1.9 *n* = 11
Granular	27.0 ± 1.2 *n* = 11	25.9 ± 1.3 *n* = 11	24.4 ± 1.3 *n* = 11	27.4 ± 0.9 *n* = 11	28.5 ± 1.1 *n* = 11	30.1 ± 1.1 *n* = 11	27.4 ± 2.1 *n* = 11	29.0 ± 1.0 *n* = 11	27.5 ± 0.8 *n* = 11
Infragranular	31.1 ± 1.0 *n* = 16	30.3 ± 1.3 *n* = 16	31.7 ± 1.3 *n* = 16	31.9 ± 0.7 *n* = 16	32.4 ± 1.6 *n* = 16	37.4 ± 1.8 *n* = 16	29.4 ± 0.9 *n* = 16	34.2 ± 1.0 *n* = 16	29.2 ± 0.7 *n* = 16

### IGF-I facilitates stimulus detection

**Figure 7.**
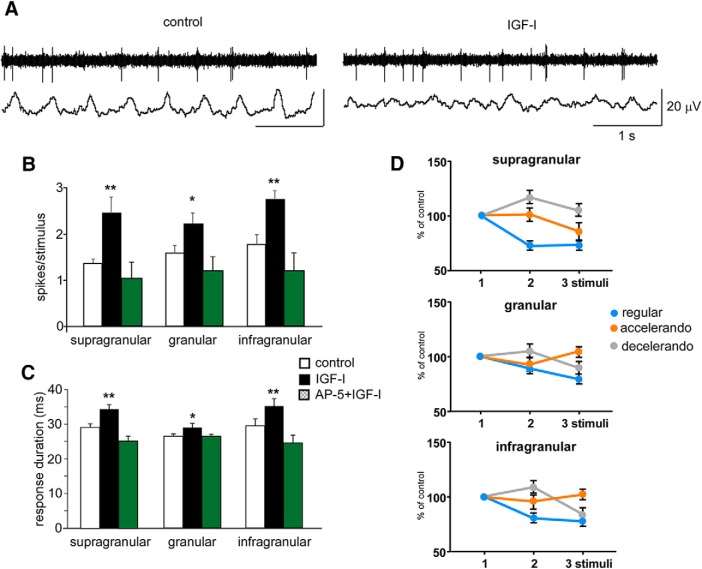
Changes in the response adaptation induced by the stimulation pattern are also observed during periods of EEG activation. ***A***, Unit and field potential activities (top and bottom traces, respectively) are shown in a representative case recorded in the supragranular layer. Intraperitoneal injection of IGF-I (1 μg/g body weight) induced a reduction of slow waves and an increase in the spontaneous firing rate. ***B***, Responses to whisker stimuli are increased after IGF-I intraperitoneal injection (closed bars) respect to control values (white bars) in supragranular (*n* = 6), granular (*n* = 7), and infragranular (*n* = 9) neurons. Facilitation by IGF-I of whisker responses is blocked after AP5 (50 μm; 0.1 μl) injection in the BC (green bars). ***C***, IGF-I injection also induces an increase of the response duration (closed bars). Similarly, the increase in the response duration evoked by IGF-I is blocked if AP5 (50 μm; 0.1 μl) is injected previously (green bars). ***D***, Mean percentage of the response respect to the control stimulus (first stimulus of the stimulation train) is shown. Response adaptation to the regular stimulation pattern is reduced and the facilitation of responses during the application of the accelerando or decelerando stimulation patterns are enhanced in supragranular, granular and infragranular layers after IGF-I administration. *p < 0.05; **p < 0.01. *Figure Contributions:* Angel Nuñez performed the experiments. All authors analyzed the data.

Above results have been obtained under urethane anesthesia, a state characterized by the prevalence of synchronous slow oscillations (Steriade, 1993; Castro-Alamancos and Bezdudnaya, 2015). However, natural active whisking occurs in aroused animals exploring the environment; a state characterized by the absence of slow oscillations (cortical activation). It has been demonstrated that systemic application of IGF-I increases neuronal firing (Carro et al., 2000; Nuñez et al., 2003; Mysoet et al., 2015) and activates the electroencephalogram (Trueba-Sáiz et al., 2013). Thus, IGF-I was injected intraperitoneally to study whisker responses and the stimulation pattern effect during a state of increased cortical activation such as occurs during anesthesia. We performed simultaneous unit and field potential recordings in the BC of urethane anesthetized mice (*n* = 8) in control conditions (after injection of 0.1 ml of saline solution, i.p.) and 20 minutes after intraperitoneal injection of IGF-I (1 μg/g body weight dissolved in 0.1 ml of saline solution). Field potentials were recorded to determine that IGF-I induced cortical activation. In control conditions, field potentials showed slow waves evoked by the anesthetic (Fig. 7*A*, control). Intraperitoneal administration of IGF-I elicited an increase of the firing rate and a reduction of the slow wave amplitude in the cortex (Fig. 7*A*, IGF-I). This effect was accompanied with an increase in the response to whisker stimulation in neurons located in all cortical layers (Fig. 7*B*, closed bars). Supragranular neurons increased whisker responses from 1.4 ± 0.08 spikes/stimulus in control condition (*n* = 6) to 2.5 ± 0.35 spikes/stimulus 20 min after IGF-I intraperitoneal injection (*p* = 0.0128; *n* = 6; paired *t* test). Similarly, granular neurons increased whisker responses from 1.6 ± 0.16 spikes/stimulus in control condition to 2.2 ± 0.24 spikes/stimulus (*p* = 0.0293; *n* = 6; paired *t* test) and infragranular neurons increased whisker responses from 1.8 ± 0.22 spikes/stimulus in control condition to 2.7 ± 0.2 spikes/stimulus after IGF-I injection (*p* = 0.0049; *n* = 9; paired *t* test). The evoked facilitation by IGF-I of whisker responses was blocked if AP5 (50 μm; 0.1 μl) was injected in the BC 10 min before IGF-I injections (Fig. 6*B*, green bars). In this condition, supragranular neurons showed 1.0 ± 0.3 spikes/stimulus (*p* > 0.05, respect to control values; *n* = 4; paired *t* test), granular neurons showed 1.2 ± 0.29 spikes/stimulus (*p* > 0.05; *n* = 4; paired *t* test) and infragranular neurons showed 1.2 ± 0.38 spikes/stimulus after IGF-I injection (*p* > 0.05; *n* = 5; paired *t* test). Consequently, the increased whisker response evoked by IGF-I may be because of activation of NMDA receptors.

As it was expected, IGF-I injection also induced an increase of the response duration (Fig. 7*C*, closed bars). Supragranular neurons increased response duration from 29.2 ± 1.08 ms in control condition (*n* = 6) to 34.5 ± 1.3 ms 20 min after IGF-I intraperiotneal injection (*p* = 0.0128; *n* = 6; paired *t* test). Similarly, granular neurons increased whisker response from 26.5 ± 0.68 ms in control condition to 29.0 ± 1.21 ms (*p* = 0.0278; *n* = 6; paired *t* test) and infragranular neurons increased whisker response from 29.5 ± 2.02 to 35.2 ± 2.15 ms after IGF-I injection (*p* < 0.001; *n* = 9; paired *t* test). Similarly, the increase in the response duration evoked by IGF-I was blocked if AP5 (50 μm; 0.1 μl) was injected in the BC 10 min before IGF-I injections (Fig. 7*C*, green bars). In this condition, supragranular neurons showed a response duration after IGF-I injection of 25.3 ± 1.44 ms (*p* > 0.05, respect to control values; *n* = 4; paired *t* test), granular neurons showed 26.5 ± 0.58 ms (*p* > 0.05; *n* = 4; paired *t* test) and infragranular neurons showed 24.6 ± 2.22 ms after IGF-I injection (*p* > 0.05; *n* = 5; paired *t* test).

In addition, response adaptation to the regular stimulation pattern was reduced and the facilitation of responses during the application of the accelerando or decelerando stimulation patterns was enhanced in all cortical layers after IGF-I administration (Fig. 7*D*), suggesting that response adaptation and detection of different timings were facilitated during a cortical activation state induced, in this case, by IGF-I.

## Discussion

During exploration, rodents move their whiskers rhythmically across objects or surfaces (Carvell and Simons, 1990; Berg and Kleinfeld, 2003; Grant et al., 2009). This rhythmic behavior induces spike responses in BC neurons that displayed a progressive adaptation with time (Derdikman et al., 2006; Katz et al., 2006). The rhythmic behavior to explore the environment is not exclusive of the somatosensory system or the whisker system, also appears in the visual or auditory systems and in different species, suggesting that it may facilitate the detection of stimuli (Szwed et al., 2003; Kleinfeld et al., 2006; Diamond and Arabzadeh, 2013). The effect of anesthesia on the adaptation, following the results reported for an oddball paradigm in anesthetized and freely-moving rats (Eriksson and Villa, 2005), suggests that response adaptation in un-anesthetized animals is likely to be much larger than any observed modification during anesthesia. Our results show that response adaptation was reduced in BC layers when a modification of the stimulation interval was introduced in the stimulation sequence (shifted stimulus), decreasing (decelerando) or increasing (accelerando) the interval between the first and the second stimuli. The response to the shifted stimulus was greater than estimated during the 4 Hz regular stimulation. This facilitation was mediated by activation of NMDA receptors since the effect was blocked by AP5. Facilitation of the shifted stimulus increased during periods of EEG activation induced by IGF-I application, probably by activation of NMDA receptors because the increment of whisker responses induced by IGF-I was also blocked by AP5. This process may contribute to improve the detection of “unexpected” stimuli that disturbed the rhythmic behavior of exploration.

Adaptation, the reduction of the response to ongoing or repeated stimulation, occurs across species and sensory modalities (Ahissar et al., 2000; Maravall et al., 2007; Kheradpezhouh et al., 2017). The functional role of the response adaptation process may be to alter the sensitivity of neurons to encode sensory stimuli more efficiently (Sharpee et al., 2006; Maravall et al., 2007, 2013; Ganmor et al., 2010) or to improve the detectability of rare stimuli by decreasing responses to frequent stimuli (Dragoi et al., 2002; Ulanovsky et al., 2003). In the somatosensory system, most studies observed response depression in different layers of BC to whisker stimulation (Ahissar et al., 2000, 2001; Martin-Cortecero and Nuñez, 2014; Kheradpezhouh et al., 2017). We also observed a response depression when the regular stimulation pattern was applied. Our findings also showed that the introduction of a temporal jitter increased the response to the shifted stimuli in all cortical layers. An increased response to irregular stimulation has been previously described in the BC, suggesting that may contribute to the detection of relevant stimuli (Lak et al., 2008; Kheradpezhouh et al., 2017).

Our findings showed that adaptation changed during the stimulation time. If we compared the response of the first stimulation trains with the last stimulation trains, we observed a reduction of adaptation with time, mainly in supragranular and infragranular layers (Fig. 4*A*). In addition, the response facilitation of the shifted stimulus was also reduced along the stimulation time (Fig. 4*B*,*C*), indicating that it may contribute to the detection of unexpected and non-repetitive stimuli.

During quiescent states the sensory adaptation observed in the neocortex is high and may be a consequence of depression at thalamocortical pathways; during activated states animals are alert exploring the environment; in this case sensory adaptation is reduced (Castro-Alamancos, 2004). Results present here also showed a reduction of sensory adaptation during activated states (in this case induced by IGF-I injection; compare Figs. 2 and 7 during the application of the regular stimulation pattern). The reduction was more evident in supragranular and infragranular cells as was indicated early (Ahissar et al., 2001). Changes in sensitivity during adaptation may allow the neuronal circuit to respond optimally to a larger range of stimuli despite the limited dynamic range of neuronal firing (Derdikman et al., 2006; Katz et al., 2006). Synaptic mechanisms, such as enhancement of inhibition (Dealy and Tolhurst, 1974) or depression of excitatory synapses (Adorján et al., 1999; Ahissar et al., 2000; Sanchez-Vives and Mccormick, 2000; Chung et al., 2002; Castro-Alamancos, 2004; Higley and Contreras, 2006; Díaz-Quesada and Maravall, 2008) have been proposed as mechanisms for adaptation. *In vivo* experiments have ruled out enhanced inhibition since iontophoretic application of GABA_A_ receptor antagonists or agonists did not affect adaptation (Martin-Cortecero and Nuñez, 2014; Ayala and Malmierca, 2018). Derdikman et al. (2006) demonstrated that responses to frequent stimuli can depress, remain unchanged, or facilitate, depending on the information conveyed by the stimulus and the cortical layer. Here, a change in the interval between stimuli is enough to facilitate the response to the shifted stimulus. Thus, these results support the notion that cortical adaptation is not an automatic mechanism during repetitive stimulation but rather a process that depends and changes according to the sensory input pattern.

Recordings of thalamic cells showed a small adaptation during the application of a regular stimulation train, consistent with other previous studies conducted on VPM or POm neurons (Hellweg et al., 1977; Ahissar et al., 2000; Ahissar and Zacksenhouse, 2001; Sosnik et al., 2001; Hartings et al., 2003; Khatri et al., 2004; Martin-Cortecero and Nuñez, 2014). Moreover, either VPM or POm thalamic neurons were not sensitive to a change in the interval between stimuli, suggesting that this property is generated, at least in part, in the BC. Other brain structures may also contribute to the detection of relevant stimuli. For example, acetylcholine could enhance sensory detection, processing, and plasticity with the intervention of complex synaptic interactions (Ego-Stengel et al., 2001; Castro-Alamancos, 2009; Rahman and Berger, 2011; Nuñez et al., 2012; Barros-Zulaica et al., 2014).

A likely mechanism for facilitation when the interval was shorter (125 ms) may be a paired-pulse facilitation mediated by the activation of NMDA receptors (Thomson, 2000; Dan and Poo, 2004; Citri and Malenka, 2008). Many long-term modifications in synaptic efficacy in the cortex have been proposed to be mediated by the activation of NMDA receptors and to be the cellular basis of the learning machinery and the detection of relevant stimuli (Nuñez et al., 2012; Nabavi et al., 2014; Gruart et al., 2015; Díez-García et al., 2017). For example, antagonists of NMDA receptors block cortical plasticity after electrical thalamic stimulation (Heynen and Bear, 2001) or whisker repetitive stimulation (Barros-Zulaica et al., 2014). Indeed, the facilitation observed during the accelerando or decelerando stimulation pattern of the shifted stimulus was blocked after cortical application of the NMDA receptor antagonist AP5 in supragranular and infragranular layers. However, this facilitation was not affected by AP5 in granular neurons during the application of the accelerando stimulation pattern, probably because of the different functional roles of each layer (Feldmeyer et al., 2013), in which different NMDA receptors subtypes are involved (Kaczmarek et al., 1997; Scheppach, 2016). Thus, the detection of an object that is interposed during the rhythmic whisker exploration may be facilitated by a mechanism that implies activation of NMDA receptors, mainly in supragranular and infragranular neurons. Granular cells express less this effect may be because they receive an important synaptic input from the thalamus, that is not sensitive to a change in the interval between stimuli, and faithfully follow the stimulation pattern, regardless of its temporal pattern.

A reduction of response adaptation to the second stimulus was also observed when the accelerando stimulation pattern was applied in comparison with the regular stimulation pattern. In this case, the interval between the first and the second stimuli was increased to 375 ms in which a facilitation mediated by NMDA receptors would be difficult to admit. However, this facilitation was also blocked by AP5 except in the case of granular cells. The increase of the interval between stimuli from 250 to 375 ms can increase the synaptic current available that can be activated by the second stimulus. In fact, previous studies have shown that at low stimulation frequencies BC neurons evoked greater responses than high stimulation frequencies (Ahissar et al., 2001; Ahissar and Zacksenhouse, 2001). In addition, membrane potential recordings of neurons in the somatosensory cortex showed that a sensory stimulus may induce depolarizations that lasted hundreds of milliseconds (Yamashita and Petersen, 2016). Thus, AP5 could reduce part of this synaptic current, blocking the facilitation observed during the application of the accelerando stimulation pattern in control conditions. Additional experiments are required to investigate the synaptic and network mechanisms responsible for this response facilitation.

It is known that systemic application of IGF-I increases neuronal firing (Carro et al., 2000; Nuñez et al., 2003; Mysoet et al., 2015) and induces EEG activation (Nishijima et al., 2010; Trueba-Sáiz et al., 2013). IGF-I is well known to be expressed throughout the brain, including the somatosensory cortex (Rotwein et al., 1988; Yamaguchi et al., 1990). Here, we show that IGF-I induced an increase in whisker responses by the activation of NMDA receptors because it was blocked by previous injection of AP5 in the BC. During the activation state evoked by IGF-I, BC neurons detected the change in the interval between stimuli, suggesting that the mechanism of detection of shifted stimulus may play a role in sensory processing during anesthesia, and also during activate states.

It is known that alterations of the rhythmic stimulation pattern induce important changes in the response (Perkel et al., 1963; Segundo et al., 1963; Villa et al., 2007), indicating that the discharge pattern of a train of spikes carries much more information than the simple mean discharge. Our results show that such higher-order information processing is likely to be primarily the outcome of an intrinsic cortical processing, built up on ascending thalamic input unaffected by stimulus dynamics. Consequently, the rhythmic exploration behavior observed in many sensory systems (Szwed et al., 2003; Kleinfeld et al., 2006; Diamond and Arabzadeh, 2013), instead of diminishing sensibility by the induction of response adaptation, may facilitate the detection of stimuli that appear surprisingly during the exploration by the activation of cortical properties.

## Synthesis

Reviewing Editor: Quentin Pittman, University of Calgary Decisions are customarily a result of the Reviewing Editor and the peer reviewers coming together and discussing their recommendations until a consensus is reached. When revisions are invited, a fact-based synthesis statement explaining their decision and outlining what is needed to prepare a revision will be listed below. The following reviewer(s) agreed to reveal their identity: Taylor Chomiak.

In general, this study appears technologically sound and is relatively straightforward. The advance to the field, in this reviewer's opinion, is incremental. However, the manuscript provides valuable insight about cortical adaptation and sensory coding of regular/irregular stimuli. The reviewers have a number of comments to improve the manuscript:

Major:

1. The is the most important point to be addressed is to provide clarity on statistics. First, only p-values are reported. Complete test statistics should be reported. Second, and related to the first point, it is unclear which statistical test was used where (e.g., what are the asterisks in Fig. 2 from?), and whether paired and unpaired t-tests were used appropriately. Second, please justify why a a chi-squared test should be used to compare proportions here and clarify where it was used, to enable the reviewers to better evaluate this.

A number of other comments relate to the clarity of presentation and interpretation of your results:

1. I am not sure that the findings are entirely surprising. As such, the authors may want to revise the title of the study, or revise the manuscript to convince the reader that it is indeed surprising.

2. The authors suggest that this facilitated response related to non-regular stimuli may play an important role in sensory processing (presumably in a physiological context). The authors would therefore need to comment on how their methodology (averages over 100 times) would relate to the physiological situation.

3. Why was AP5 injected at a single location (i.e., 300 μm deep in the BC)?

4. The most important point to be addressed in my opinion is the statistics. First, only p-values are reported. Complete test statistics should be reported. Second, and related to the first point, it is unclear which statistical test was used where (e.g., what are the asterisks in Fig. 2 from?). Second, I am not sure a chi-squared test should be used to compare proportions here. But again, I am not sure where it was used, so it is difficult to evaluate this.

5. The authors may want to comment of the fact that even though they are recording the supragranular layers, for example, how do they know they are recording from supragranular neurons rather than infragranular-neuron distal dendrites (e.g., Moore et al. (2017) Dynamics of cortical dendritic membrane potential and spikes in freely behaving rats. Science DOI: 10.1126/science.aaj1497).

6. On page 8 the authors claim that “Layer 5 neurons showed a higher response during the application of the ‘accelerando’ stimulation pattern”. But how do they know that these are layer 5 neurons?

7. On page 8, the authors also state: “suggesting that the detection in the stimulation pattern observed in the BC was due to a cortical mechanism.” This reviewer is not sure that he agrees with this statement in its entirety as sub-cortical acetylcholine projections to the cortex may be playing an important role as well (e.g., 11. Ego-Stengel et al. (2001) Acetylcholine-dependent induction and expression of functional plasticity in the barrel cortex of the adult rat. J Neurophysiol. Jul;86(1):422-37). In fact, acetylcholine can lead to after-depolarization in neurons in many areas of the cortex (e.g., Rahman J, Berger T (2011) Persistent activity in layer 5 pyramidal neurons following cholinergic activation of mouse primary cortices. Eur J Neurosci 34: 22-30).

8. A few minor issues [my emphasis added]: “The recording session lasted for 4-5 hours after the BEGIN of the surgical procedure”; “The effect of the temporal pattern in a spike train has been STUDIED LONG TIME AGO by Segundo and colleagues”; page 10 ‘Fig. 6B, green bars’ - there are no green bars?

9.The authors apply a train of 3 air-puffs to whiskers either with a ‘regular’ pattern (at 250ms intervals) or with different intervals (‘decelerando’: 125-375ms or ‘accelerando’ 375-125ms). The authors find that across all cortical layers, neurons exhibit less adaptation to the stimuli containing temporal jitter. It is important to note that at the time of the second air-puff the only difference between the ‘regular’, ‘accelerando’ and ‘decelerando’ is simply in the frequency of stimulation. As such, at this point in time, the 250ms interval is not any more regular than the shorter or longer intervals. The differentiation of stimulus trains into regular, accelerando and decelerando emerges at the time of the 3rd air-puff. As indicated in the Methods section and schematically shown in Figure 1, the regular, accelerando and decelerando trains are not intermixed but are each repeated 100 times in separate blocks. It is therefore likely that the effect of regularity is observed at the time of the second air-puff due to this block design. It would help the reader if the authors make this point more clear (it took me a while to understand this), and also try to quantify how quickly the enhanced adaptation to regular stimulation develops within the regular block.

10. It is not clear to me if the authors record from the same neurons before and after the application of AP5. I understand from the number of neurons recorded in each condition that this is not the case for all recordings. However, if there are neurons in the data set that have been maintained before and after application of AP5, then it would be interesting to show more raw data for these neurons to better visualize the changes in response profiles induced by AP5.

11. The authors indicate that “A likely mechanism for facilitation when the interval was shorter (125ms) may be a pair pulse facilitation mediated by the activation of NMDA receptors (Thomson, 2000, Dan and Poo, 2004, Citri and Malenka, 2008).”

However, the facilitation is observed both for the shorter (125ms) and the longer interval (375ms). The results of the AP5 application are interesting, and do suggest a role for NMDA receptors. However, the authors need to revise the text to give a more consistent logic for the NMDA experiments.

12. The data presented in the right column of Figure 2 is confusing. What is ‘% of regular response’? If I understand the Figure correctly, this is plotting the difference from regular. Please use a more clear y label. Is this with respect to the response to the first stimulus in the regular condition? Also, the very first two data points (on stimulus #1) seem to be set to zero instead of reflecting the actual differences in the collected data. (I understand that the response to the first air-puff might be expected to be similar across the regular, accelerando and decelerando stimuli. However, the actual responses recorded are not expected to be absolutely identical.)

13. A number of previous research articles have investigated the response of barrel cortex neurons to a series of regular/irregular deflections and these should be included in the Discussion section (please see Fig. 6 in Kheradpezhouh et al, 2017, Scientific Reports; and Lak et al, 2007 Cerebral Cortex). The findings reported here are consistent with the previous literature in showing that temporal noise reduces the adaptation in a majority of neurons and enhances the overall response to stimuli. The current manuscript provides valuable insight beyond those previous publications, including identifying the origin of the observation in cortex (by recording from thalamic nuclei), studying the laminar organization of the response profiles, as well as experiments that test the involvement of NMDA receptors in the observed phenomenon.

14. There are a number of typos in the manuscript that should be corrected by a more careful proof reading. Please see the following examples:

Page 3: may content information → may contain information

Page 6: was calculated respect to → with respect to

Page 7: stimulation pattern respect to the corresponding → relative to the

Page 8: to establish if this mechanism is proper of the cortex

Page 9: suggesting that the detection in the stimulation pattern observed in the

Page 9: In this case, response duration was measured in the first stimulus of the

stimulation train. → Did you mean “was measured relative to the first stimulus”?

## Author Response

Synthesis Statement for Author (Required):

In general, this study appears technologically sound and is relatively straightforward. The advance to the field, in this reviewer's opinion, is incremental. However, the manuscript provides valuable insight about cortical adaptation and sensory coding of regular/irregular stimuli. The reviewers have a number of comments to improve the manuscript:

We sincerely thank the Reviewers for their helpful comments and suggestions for improvement on the previous version of the manuscript. We have included new data on the response adaptation during the 100 stimulation trains (new Figure 4), as well as a new Table 2 to include the values of the response duration in control conditions. Besides, we have made numerous minor changes throughout the text to improve clarity or to correct grammar/typing errors.

Major:

1. This is the most important point to be addressed is to provide clarity on statistics. First, only p-values are reported. Complete test statistics should be reported. Second, and related to the first point, it is unclear which statistical test was used where (e.g., what are the asterisks in Fig. 2 from?), and whether paired and unpaired t-tests were used appropriately. Second, please justify why a chi-squared test should be used to compare proportions here and clarify where it was used, to enable the reviewers to better evaluate this.

We have indicated in this new version the t-test used in each case. Also, we agree with the reviewers (see below) that the chi-squared test is not appropriate for our data. Thus, we have analyzed the data using paired and unpaired t-tests on spike responses instead of on the percentages.

A number of other comments relate to the clarity of presentation and interpretation of your results:

1. I am not sure that the findings are entirely surprising. As such, the authors may want to revise the title of the study, or revise the manuscript to convince the reader that it is indeed surprising.

The title has been changed.

2. The authors suggest that this facilitated response related to non-regular stimuli may play an important role in sensory processing (presumably in a physiological context). The authors would therefore need to comment on how their methodology (averages over 100 times) would relate to the physiological situation.

We agree that a stimulation block of 100 applications of the stimulation train is not a physiological situation although let us to reveal the mechanism of detection non-regular stimuli. Thus, we have compared the whisker responses of the first 25 stimulation trains with the last 25 stimulation trains during the 100 stimulation train block to study if adaptation affect response facilitation of non-regular stimuli. We have included these findings in a new Figure 4 and in the text (page 9, first paragraph) to show that the detection was higher at the beginning of the stimulation block and was reduced at the end of the stimulation block.

3. Why was AP5 injected at a single location (i.e., 300 μm deep in the BC)?

We applied AP5 only at 300 μm deep because 0.1 μl is enough to affect all cortical layers around the electrode. To illustrate this point, we have included in Figure 5 an inset with a PSTH in control condition and after AP5 injection to show the reduction of whisker responses in a representative layer 5 neuron.

4. The most important point to be addressed in my opinion is the statistics. First, only p-values are reported. Complete test statistics should be reported. Second, and related to the first point, it is unclear which statistical test was used where (e.g., what are the asterisks in Fig. 2 from?). Second, I am not sure a chi-squared test should be used to compare proportions here. But again, I am not sure where it was used, so it is difficult to evaluate this.

As is indicated above (Major point), we concur with reviewers that the chi-squared test is not appropriate for our data. Data were normally distributed according to the Shapiro-Wilk normality test thus, we have analyzed the data using paired and unpaired t-tests on spike responses instead of on the percentages. This change is indicated in the Statistical Analysis Section of Material and Methods (page 6, last paragraph).

5. The authors may want to comment of the fact that even though they are recording the supragranular layers, for example, how do they know they are recording from supragranular neurons rather than infragranular-neuron distal dendrites (e.g., Moore et al. (2017) Dynamics of cortical dendritic membrane potential and spikes in freely behaving rats. Science DOI: 10.1126/science.aaj1497).

We have indicated (page 7, first paragraph) that the low spontaneous firing rate showed for neurons included in this study discarded that these recordings could be performed in pyramidal dendrites because they show firing rates larger than somatic spike rates (Moore et al., 2017). Also, our records showed spikes of negative polarity and fast slope what is also against the dendritic spikes. Thank you for the reference that has been included in this new version.

6. On page 8 the authors claim that “Layer 5 neurons showed a higher response during the application of the ‘accelerando’ stimulation pattern”. But how do they know that these are layer 5 neurons?

As is indicated in Methods, following the recording session, electrolytic lesions were performed at the top and at the bottom of the electrode array for subsequent histological reconstruction of the electrode tracks in coronal sections through the barrel cortex (page 5, last paragraph). The deep of each recorded neuron indicated the cortical layer in which it was recorded.

7. On page 8, the authors also state: “suggesting that the detection in the stimulation pattern observed in the BC was due to a cortical mechanism.” This reviewer is not sure that he agrees with this statement in its entirety as sub-cortical acetylcholine projections to the cortex may be playing an important role as well (e.g., 11. Ego-Stengel et al. (2001) Acetylcholine-dependent induction and expression of functional plasticity in the barrel cortex of the adult rat. J Neurophysiol. Jul;86(1):422-37). In fact, acetylcholine can lead to after-depolarization in neurons in many areas of the cortex (e.g., Rahman J, Berger T (2011) Persistent activity in layer 5 pyramidal neurons following cholinergic activation of mouse primary cortices. Eur J Neurosci 34: 22-30).

We agree with the reviewer that acetylcholine may induce functional plasticity in the cortex. Indeed, we have published several works indicating that ACh may switch the response of L5 pyramidal neurons from a tonic mode to a bursting mode in vitro, causing a potent and sustained response enhancement with possible consequences for the function of the barrel cortex (Nuñez et al. 2012; Diez-Garcia et al. 2017). Also, this cholinergic-facilitation was observed in vivo in anesthetized rats (Barros-Zulaica et al. 2014). Thus, we have removed this sentence from the Results Section and we have deal with this matter in the Discussion Section (page 14, first paragraph). Thank you for references that have been included in this new version.

8. A few minor issues [my emphasis added]: “The recording session lasted for 4-5 hours after the BEGIN of the surgical procedure”; “The effect of the temporal pattern in a spike train has been STUDIED LONG TIME AGO by Segundo and colleagues”; page 10 ‘Fig. 6B, green bars’ - there are no green bars?

These suggestions have been included in this new version.

9.The authors apply a train of 3 air-puffs to whiskers either with a ‘regular’ pattern (at 250ms intervals) or with different intervals (‘decelerando’: 125-375ms or ‘accelerando’ 375-125ms). The authors find that across all cortical layers, neurons exhibit less adaptation to the stimuli containing temporal jitter. It is important to note that at the time of the second air-puff the only difference between the ‘regular’, ‘accelerando’ and ‘decelerando’ is simply in the frequency of stimulation. As such, at this point in time, the 250ms interval is not any more regular than the shorter or longer intervals. The differentiation of stimulus trains into regular, accelerando and decelerando emerges at the time of the 3rd air-puff. As indicated in the Methods section and schematically shown in Figure 1, the regular, accelerando and decelerando trains are not intermixed but are each repeated 100 times in separate blocks. It is therefore likely that the effect of regularity is observed at the time of the second air-puff due to this block design. It would help the reader if the authors make this point more clear (it took me a while to understand this), and also try to quantify how quickly the enhanced adaptation to regular stimulation develops within the regular block.

We agree with the reviewer that the stimulation pattern may be confuse. As you indicate, we compare a regular stimulation pattern at 4 Hz with a stimulation pattern containing temporal jitter. This pattern of stimulation is reminiscent of that produced by an object that interferes the rhythmic movement of whiskers during the whisking behavior. We now make this point explicit in page 5.

We repeated 100 times the stimulation pattern and we showed the average response facilitating the detection of response changes. Now, we include a new figure (Fig. 4) to compare the response to the first with the last 25 blocks of stimuli. In this figure we see that adaptation to 4 Hz regular stimulation and response facilitation were greater in the first stimulation trains than in the last stimulation trains (page 9, first paragraph).

10. It is not clear to me if the authors record from the same neurons before and after the application of AP5. I understand from the number of neurons recorded in each condition that this is not the case for all recordings. However, if there are neurons in the data set that have been maintained before and after application of AP5, then it would be interesting to show more raw data for these neurons to better visualize the changes in response profiles induced by AP5.

In the previous version we had included a larger number of cells in the control group that neurons that were studied later during the application of AP5 with different stimulation patterns. Now, we only include the data set of neurons that have been maintained before and after application of AP5 (page 9).

11. The authors indicate that “A likely mechanism for facilitation when the interval was shorter (125ms) may be a pair pulse facilitation mediated by the activation of NMDA receptors (Thomson, 2000, Dan and Poo, 2004, Citri and Malenka, 2008).”

However, the facilitation is observed both for the shorter (125ms) and the longer interval (375ms). The results of the AP5 application are interesting, and do suggest a role for NMDA receptors. However, the authors need to revise the text to give a more consistent logic for the NMDA experiments.

The effect of the AP5 on the facilitation at intervals of 375 ms was also surprising for us. It is possible that whisker stimulation did not activates synchronically barrel cortical neurons and thus, glutamatergic synaptic currents could summate and remain activated at that long intervals. In agreement with that, membrane potential recordings of neurons in the somatosensory cortex showed that a sensory stimulus may induce depolarizations that lasted hundreds of milliseconds (Yamashita and Petersen). Moreover, an increase of the interval between stimuli (from 250 ms to 375 ms) may increase the synaptic current that can be activated. In fact, previous studies have shown that at low stimulation frequencies barrel cortical neurons displayed greater responses (e.g. Ahissar et al. 2001). Thus, AP5 could reduce part of this synaptic current, blocking the facilitation observed during the application of the accelerando stimulation pattern. We point out this issue in the discussion (pages 14-15).

12. The data presented in the right column of Figure 2 is confusing. What is ‘% of regular response’? If I understand the Figure correctly, this is plotting the difference from regular. Please use a more clear y label. Is this with respect to the response to the first stimulus in the regular condition? Also, the very first two data points (on stimulus #1) seem to be set to zero instead of reflecting the actual differences in the collected data. (I understand that the response to the first air-puff might be expected to be similar across the regular, accelerando and decelerando stimuli. However, the actual responses recorded are not expected to be absolutely identical.)

Following the Reviewer suggestion, we have changed the label of these plots. The plots shown on the right display differences in the percentage of responses to the 1-3 stimuli during the application of the ‘accelerando’ or ‘decelerando’ stimulation pattern respect to the corresponding percentage to 1-3 stimuli during the application of a regular stimulation pattern. We considered the response of the first stimuli as the 100%. For this reason, there is not differences between percentages of responses to the application of different stimulation patterns although the actual values of response were not equal. However, there was differences in the response percentages in the following stimuli (2-3). Also, we have change the legend of this figure (Fig. 2) to clarify this point.

13. A number of previous research articles have investigated the response of barrel cortex neurons to a series of regular/irregular deflections and these should be included in the Discussion section (please see Fig. 6 in Kheradpezhouh et al, 2017, Scientific Reports; and Lak et al, 2007 Cerebral Cortex). The findings reported here are consistent with the previous literature in showing that temporal noise reduces the adaptation in a majority of neurons and enhances the overall response to stimuli. The current manuscript provides valuable insight beyond those previous publications, including identifying the origin of the observation in cortex (by recording from thalamic nuclei), studying the laminar organization of the response profiles, as well as experiments that test the involvement of NMDA receptors in the observed phenomenon.

Thank you very much for your comments. We have included these paper in the Discussion because they provide consistent and complementary results (page 13).

14. There are a number of typos in the manuscript that should be corrected by a more careful proof reading. Please see the following examples:

Page 3: may content information → may contain information

Page 6: was calculated respect to → with respect to

Page 7: stimulation pattern respect to the corresponding → relative to the

Page 8: to establish if this mechanism is proper of the cortex

Page 9: suggesting that the detection in the stimulation pattern observed in the

Page 9: In this case, response duration was measured in the first stimulus of the

stimulation train. → Did you mean “was measured relative to the first stimulus”?

All typos have been corrected.

Response duration was measured for each stimulus into the stimulation train during the application of different stimulation patterns, as is indicated now at the end of the third paragraph, page 6.
